# Alexithymic and autistic traits differentially predict personality disorder dimensions

**DOI:** 10.1177/13623613251338650

**Published:** 2025-06-01

**Authors:** Laura Albantakis, Leonie Weindel, Marie-Luise Brandi, Imme C. Zillekens, Lara Henco, Hanna Thaler, Lena Schliephake, Leonhard Schilbach

**Affiliations:** 1 Max Planck Institute of Psychiatry, Munich, Germany; 2 International Max Planck Research School for Translational Psychiatry, Munich, Germany; 3 Department of Psychiatry and Psychotherapy, Ludwig Maximilians University, Munich, Germany; 4 University Medical Center Freiburg, Germany; 5 University of Muenster, Germany; 6 Department of General Psychiatry 2, LVR-Klinikum Duesseldorf/Kliniken der Heinrich-Heine-Universitaet, Duesseldorf, Germany

**Keywords:** alexithymia, autism, personality disorder dimensions

## Abstract

Alexithymia and autism are variably characterized by social cognitive and perceptual deficits, which can lead to profound social interaction difficulties. Such difficulties are also the hallmark of personality disorders (PDs), but the potential link between alexithymia, autism, and PDs remains unclear. Here, we investigated whether autistic and/or alexithymic traits are associated with specific PD dimensions as measured by the Assessment of Diagnostic and Statistical Manual of Mental Disorders, Fourth Edition (*DSM*-IV) Personality Disorders Questionnaire (ADP-IV). We included individuals with a formal autism diagnosis (AP, *n* = 89), individuals with a psychiatric diagnosis other than autism (NAP, *n* = 51), and neurotypical participants (NPs, *n* = 84). We applied linear regression analyses with autistic and alexithymic traits as predictors and PD dimensions as outcome variables, while controlling for age, depressive, and socially anxious symptoms. While autistic traits predicted the obsessive-compulsive PD dimension, alexithymic traits predicted the borderline PD dimension. Autistic and alexithymic traits were both significant predictors of the schizoid PD dimension. Taken together, our results point toward overlaps between autistic traits and specific PD dimensions that are well recognized as differential diagnoses of autism, while alexithymia was differentially linked to the borderline PD dimension.

**Lay abstract:**

Autistic people have trouble identifying their own emotions and others’ emotions. They also struggle to describe their emotions. People with alexithymia have similar difficulties. This can make it hard for autistic and alexithymic people to react appropriately to situations. Some may avoid places, while others may start overthinking. This can lead to patterns of behavior and thinking that limit us in our daily lives. If these patterns become a part of our personality, we may develop a personality disorder (PD). In our study, we looked at whether autistic and alexithymic people have certain PD dimensions. We included autistic participants, non-autistic participants with social-interactive difficulties, and non-autistic participants without social-interactive difficulties. We found that autistic features were linked to obsessive-compulsive PD dimension. Alexithymic features were linked to borderline PD dimension. Autistic and alexithymic features were linked to schizoid PD dimension. Our results point toward overlaps between autistic traits and PD dimensions that are well recognized as differential diagnoses of autism. The exception was borderline PD dimension, which was linked to alexithymic traits.

Interacting with others is essential for emotional well-being and social functioning (Xie et al., 2016). Social interaction modulates the self-perception and the perception by others with impact on the individual’s personality (Xie et al., 2016). Thus, it is not surprising that social interaction difficulties are a transdiagnostic phenomenon of various psychiatric conditions including autism, personality disorders (PDs), psychoses, affective disorders, and anxiety disorders. In light of this, it has been argued that psychiatric conditions can be construed as “disorders of social interaction” (Schilbach, 2016, 2022). In case of autism, social impairments are a defining feature of the condition, which manifest early in life and persist during the lifespan (American Psychiatric Association [APA], 2013). In other cases, constant negative and derogatory social interactions may increase maladaptive emotion-regulation strategies and the risk of the development of PDs (Howard & Cheavens, 2023; Möhler, 2022).

## Personality disorders

While every human being possesses personal quirks and flaws as part of the individual personality profile, persons with a PD show an inflexible form of thinking, functioning, and behaving with disadvantageous effects on their mental well-being (APA, 2013; Tyrer et al., 2015). More precisely, people with PD describe difficulties in interpreting social situations and interacting with others, ultimately leading to conflicts and impairments in many aspects of life including relationships, work, and living conditions (APA, 2013; Tyrer et al., 2015). In other words, social interaction difficulties and misaligned interpersonal expectations have been considered as a hallmark of PD (Henco et al., 2020; Pott & Schilbach, 2022). The Diagnostic and Statistical Manual of Mental Disorders, Fifth Edition (*DSM*-V) lists 10 different forms of PDs, which are categorized into three clusters with shared features and symptoms (APA, 2013). “Cluster A” PDs are characterized by suspicion or lack of interest in others, while “Cluster B” PDs contain dramatic, overly emotional, or unpredictable thinking and behavior. “Cluster C” PDs have anxious thinking or behavior in common (APA, 2013). The Assessment of Diagnostic and Statistical Manual of Mental Disorders, Fourth Edition (*DSM*-IV) PDs questionnaire (ADP-IV) is a self-report measure of the *DSM*-IV Axis II PDs (Schotte et al., 2004). The ADP-IV self-report questionnaire was developed to provide either a categorical or a dimensional assessment of different PD dimensions.

## Autism

Autism, or autism spectrum disorder (ASD) according to *DSM*-V, is defined as a neurodevelopmental condition with impairments in social communication and social interaction as well as restricted, repetitive patterns of behavior, interests, or activities (APA, 2013). While autism-related symptoms usually manifest in early childhood, PD-related symptoms become more evident later in life, for instance, in late adolescence or early adulthood (APA, 2013; Johnson et al., 2008; Lugnegård et al., 2012). Nevertheless, it is possible to receive an autism diagnosis later in life due to compensation mechanisms or person-environment fit for example (Albantakis, 2020; Lai & Baron-Cohen, 2015). While PDs have been considered as differential diagnoses to autism for a long time, researchers have started to consider PDs as potentially co-occurring conditions of autism (Allely et al., 2023; Barlattani et al., 2023; Fitzpatrick et al., 2023; Rinaldi et al., 2021).

## Alexithymia

A key factor in the context of “disorders of social interaction” may be alexithymia, a subclinical condition in which affected individuals have difficulties identifying and describing their own emotions (G. J. Taylor, 2000) as well as emotions in others, also leading to social interaction difficulties (R. Cook et al., 2013; Starita et al., 2018). Some researchers have suggested that social impairments seen in autistic individuals might, in fact, be due to concurrent alexithymia rather than representing a genuine feature of autism (Bird & Cook, 2013; Trevisan et al., 2016). Approximately 50% of autistic people display clinically significant levels of alexithymia (Hill et al., 2004; Kinnaird et al., 2019; Milosavljevic et al., 2016; Morie et al., 2019). However, alexithymia is also found in individuals with PDs, depression, and social anxiety for example (Dalbudak et al., 2013; Honkalampi et al., 2000; Joyce et al., 2013).

## Alexithymia, autism, and PDs

Multiple factors contribute to the development of PDs, including unconscious processes, genetic vulnerability, early childhood experiences, adverse life events, and the influence of internal conflicts (e.g. Fariba et al., 2024a). Previous attempts to better understand the co-occurrence of traditional PD categories and autism (without co-occurring intellectual impairment) have identified a mixed pattern of overlapping symptoms in PD and autism, which warrants further research (Bach & Vestergaard, 2023; Rinaldi et al., 2021). Here, we propose that taking a dimensional approach into autism, alexithymia, and PD and their potential overlaps might offer new insights. Most previous studies investigating associations between autism, alexithymia, and PDs have only included autistic or alexithymic traits but not both traits into their analyses (e.g. M. L. Cook et al., 2020; Coolidge et al., 2013; Richards et al., 2023). This gap particularly affects the four selected PD dimensions, namely schizoid, borderline, avoidant, and obsessive-compulsive PD (OCPD) dimensions, which we briefly describe in relation to autism and alexithymia.

Schizoid PD (Cluster A) is characterized by a pervasive pattern of detachment from social relationships and a restricted range of expression of emotions in interpersonal settings (Fariba et al., 2024b). People with schizoid PD show significant social withdrawal and can be viewed by others as eccentric, solitary, or isolated. They experience considerable discomfort with social interactions, and their introversion is used as a defense mechanism to avoid psychological discomfort (Fariba et al., 2024b). Autistic individuals and people with schizoid PD show similar patterns of social and communicative impairments, supporting the idea of a shared phenotype (M. L. Cook et al., 2020; Ford & Crewther, 2014). While people with schizoid PD are supposed to be more affected by deficits in social motivation, autistic individuals are thought to be more affected by deficits in social skills or capacity (M. L. Cook et al., 2020). High levels of alexithymia have been associated with schizoid PD (Coolidge et al., 2013) and autism (see section Alexithymia), indicating that both traits might be of relevance in schizoid PD.

Borderline PD (Cluster B) is characterized by a pervasive pattern of emotional lability, impulsiveness, interpersonal difficulties, identity disturbances, and disturbed cognition (e.g. mentalization deficits, depersonalization, derealization, and hallucinations; Lieb et al., 2004). Both borderline PD and autism have been associated with elevated levels of anxiety and depression (Richards et al., 2023), which might evolve from exposure to adverse events and trauma (Copeland et al., 2007; Griffiths et al., 2019). Individuals with borderline PD and autistic individuals experience traumatic events more often than people from the general population (Golier et al., 2003; J. L. Taylor & Gotham, 2016). Higher levels of alexithymia have been found in both borderline PD (New et al., 2012; Flasbeck et al., 2020) and autism (see section Alexithymia), which may be an indicator of previous unelaborated traumatic experiences and could therefore be conceptualized as a maladaptive–reactive construct (Franzoni et al., 2013).

Avoidant PD (Cluster C) is characterized by a persistent pattern of social anxiety, heightened sensitivity to rejection, and pervasive feelings of inadequacy, coupled with a deep-rooted longing for meaningful connections with others (Fariba et al., 2024c). Like autism, avoidant PD is a chronic condition with an onset at an early age and a lifelong impact (Lampe & Malhi, 2018). Autistic traits have been found to predict social avoidance and distress via perceived stress and interpersonal alienation in non-autistic people (Li et al., 2023). Independent of depressive symptoms, alexithymia has been associated with avoidant PD in a cohort of outpatients (Loas et al., 2015). However, patients with avoidant PD had less difficulty identifying their own emotions than patients with borderline PD (New et al., 2012).

OCPD (Cluster C) is characterized by an intense focus on perfection, a strong sense of order, and a rigid need for control (Diedrich & Voderholzer, 2015). Individuals with OCPD may be inflexible, often resist change, and can be overwhelmed by minute details, rules, and schedules, hindering their productivity (Rizvi & Torrico, 2023). While autism often manifests in early childhood and comprises many symptoms, including social communication and interaction challenges, OCPD typically emerges in late adolescence or early adulthood and revolves around perfectionism, orderliness, and a need for control (Rizvi & Torrico, 2023). Previous studies showed correlations between autistic traits and OCPD dimension (Abd Elgawad et al., 2022; Gadelkarim et al., 2019) as well as between alexithymic traits and OCPD dimension (De Panfilis et al., 2015).

In a previous study, we found evidence that alexithymia might be a vulnerability factor for developing depression and social anxiety in autistic and non-autistic participants (Albantakis et al., 2020), which had been supported by recent studies (Bloch et al., 2021; Mason & Happé, 2022). In the present study, we extended our previous investigation by including the investigation of PD dimensions—as measured by the ADP-IV questionnaire—representing tendencies of dysfunctional coping mechanisms, while taking age and psychiatric co-occurring conditions into account.

## Study aims

In this study, we investigated whether the degree of autistic and alexithymic traits correlated with scores on specific PD dimensions (namely schizoid, borderline, avoidant, and OCPD) across three different groups of participants. Autistic, alexithymic, and PD dimensions were assessed as dimensional constructs and are known to be present in both clinical and non-clinical populations (Austin, 2005; Fietz et al., 2018; Franz et al., 2008; Ingersoll & Wainer, 2014; Kamp-Becker et al., 2010; Rinaldi et al., 2021; Ruzich et al., 2015; Salminen et al., 1999). Thus, we extended the investigation beyond autism and included participants with social interaction difficulties due to other psychiatric conditions as well as neurotypicals without any history of psychiatric or neurological complaints from the general population. Social interaction difficulties or “disorders of social interaction” have recently been recognized as an unmet medical condition, which can be addressed by psychiatric services that focus on differential diagnoses and the social impairments associated with a given disorder (Albantakis & Schilbach, 2020; Schilbach, 2016). This approach is in concordance with a dimensional and transdiagnostic approach to mental health, as, for instance, set in the National Institute of Mental Health (NIMH) Research Domain Criteria (RDoC) framework (Insel et al., 2010). In this study, we also aimed at excluding relevant confounders by controlling for age, depressive symptoms, and socially anxious symptoms and focused on elucidating whether certain PD dimensions are linked to autism and/or alexithymia.

## Methods

### Sample

The study included three groups of participants (Table 1): autistic participants (AP), non-autistic participants with social interaction difficulties (NAP), in which autism has been diagnostically ruled out, and non-autistic participants without social interaction difficulties, also referred to as neurotypical participants (NP). All participants had at least average intelligence (IQ = 100 ± 15), which was an inclusion criterion of the study to ensure that participants were able to understand the written instructions of the study and fill out the applied questionnaires independently of third parties. Furthermore, AP and NAP were patients admitted to the Outpatient Clinic and Day Clinic for Disorders of Social Interaction at the Max Planck Institute of Psychiatry, Munich, Germany, from April 2015 to January 2018 primarily for a diagnostic evaluation of autism. Patients were tested in our neuropsychology department with “Wechsler Adult Intelligence Scale–Fourth edition” (WAIS-IV), while the neurotypical research participants were assessed by making use of the “Vocabulary IQ test” (in German: “Wortschatztest,” WST).

**Table 1.** Characteristics of study participants according to the diagnostic group.

Variables	AP	NAP	NP	*F*	*p*	*η* ^2^
*N*	89	51	84	-	-	-
Sex: female (%)	27 (30.3)	21 (41.2)	52 (61.9)	-	-	-
Alexithymia (yes/no)	52/37	25/26	5/79			-
Mean age in years (*SD*)	34.80 (10.57)	34.76 (12.44)	25.82 (6.28)^a^	36.03	<.001***	.14
Mean ADOS-2 (*SD*)	6.80 (3.15)^b^	3.83 (2.90)^c^	-	-	<.001***	.97^d^
Mean AQ (*SD*)	36.95 (7.29)	32.44 (8.92)	14.70 (5.74)	411.84	<.001***	.62
Mean TAS-20 (*SD*)	62.92 (10.04)	59.46 (10.51)	43.56 (10.67)	148.95	<.001***	.39
Mean BDI-II (*SD*)	17.63 (11.43)	22.80 (10.72)	4.92 (4.72)	79.0	<.001***	.22
Mean LSAS (*SD*)	78.26 (25.76)	71.60 (27.72)	28.40 (16.57)	196.61	<.001***	.44
Mean ADP-IV schizoid (*SD*)	25.67 (6.41)	27.04 (5.95)	14.40 (6.60)	132.54	<.001***	.33
Mean ADP-IV borderline (*SD*)	28.48 (9.79)	36.45 (10.85)	23.23 (9.64)	11.35	.001**	.04
Mean ADP-IV avoidant (*SD*)	32.42 (8.22)	33.25 (7.48)	16.57 (7.58)	174.92	<.001***	.39
Mean ADP-IV OC (*SD*)	33.47 (8.39)	33.22 (8.17)	22.96 (8.61)	66.38	<.001***	.22

AP: autistic participants; NAP: non-autistic participants with difficulties in social interaction; NP: neurotypical participants; ADOS-2: Autism Diagnostic Observation Schedule–2nd edition (overall total calibrated severity score); AQ: Autism-Spectrum Quotient (scale: 0–50); TAS-20: Toronto Alexithymia Scale-20 (scale: 20–100); alexithymia: TAS-20 scores ⩾ 61; BDI-II: Beck Depression Inventory-II (scale: 0–63); LSAS: Liebowitz Social Anxiety Scale (scale: 0–144); ADP-IV: Assessment of *DSM*-IV Personality Disorders (dimensional scores); OC: obsessive-compulsive; *SD*: standard deviation; ANOVAs: analyses of variance. ANOVAs were calculated with planned contrasts to determine group differences. Results are based on 1000 bootstrap samples.

a One missing value.

b Ten missing values.

c Fifteen missing values.

d Cohen’s *d* (unpaired *t*-test).

* *p* < .05. **p < .01. ***p < .001.

All patients received a formal diagnostic assessment of autism according to the national autism guidelines (Arbeitsgemeinschaft der Wissenschaftlichen Medizinischen Fachgesellschaften [AWMF], 2015). The diagnostic process is described in more detail elsewhere (Albantakis et al., 2020). Depending on the results of the diagnostic evaluation, patients were divided into two groups: Individuals who fully met the *DSM*-V criteria of ASD and thus received the diagnosis of ASD were grouped as AP (*n* = 89). Individuals who showed significant impairments of their social communicative skills but did not fulfill the diagnostic criteria of ASD according to *DSM*-V are referred to as NAP (*n* = 51). For example, people characterized as NAP had social interaction difficulties but did not give any indication for repetitive and restrictive patterns of behavior, interests, or activities in the present or past. We also included a third group of adults without any history of psychiatric or neurological impairments, defined as NP (*n* = 84). Subjects of this group had taken part in previous research projects of the Independent Max Planck Research Group for Social Neuroscience at the Max Planck Institute of Psychiatry, Munich, Germany, in the past. All study participants (*N* = 224) provided written informed consent. Ethical approval was granted by the Ethics Committee of the Ludwig-Maximilian-University, Munich (project number: 18-562). All procedures were performed in accordance with the Declaration of Helsinki. The diagnostic procedure of the clinical assessment for autism at the Max Planck Institute of Psychiatry has been described in detail elsewhere (Albantakis et al., 2020*)*. Community members were not involved in the study.

### Clinical data

Medical and psychosocial histories from all patients (AP and NAP) were assessed in interviews conducted by a psychologist or psychiatrist experienced in diagnosing autism (Supplemental Table S1 for co-occurring psychiatric conditions). As part of the regular diagnostic process, AP and NAP were tested using the Autism Diagnostic Observation Schedule-2 (ADOS-2; Hus & Lord, 2014) (Table 1).

### Measures

Participants of all three groups (AP, NAP, and NP) were asked to fill out the same set of psychometric questionnaires (Table 2). To evaluate autistic traits, the Autism-Spectrum Quotient (AQ) (Baron-Cohen et al., 2001) was used. The Toronto Alexithymia Scale with 20 items (TAS-20) (Bagby et al., 1994) reflected the level of alexithymia in individuals, whereas the Liebowitz Social Anxiety Scale (LSAS) (Fresco et al., 2001) and Beck Depression Inventory-II (BDI-II) (Beck et al., 1996) provided information about symptoms of social anxiety and depression, respectively. To evaluate PD dimensions, participants filled out the Assessment of *DSM*-IV Personality Disorders (ADP-IV) by Schotte et al. (1998). Only data sets with less than 10% of missing data per instrument were included (Table 1).

**Table 2.** Overview of psychometrics of primary study measures.

Psychometric instrument	Abbreviation	Assessment of	Scale
Autism-Spectrum Quotient	AQ	autistic traits	0–50
Assessment of *DSM*-IV Personality Disorders	ADP-IV	personality disorder dimension	0–25*
Beck Depression Inventory-II	BDI-II	depressive symptoms	0–63
Liebowitz Social Anxiety Scale	LSAS	social anxious symptoms	0–144
Toronto Alexithymia Scale with 20 items	TAS-20	alexithymic traits	20–100

* Varying dimensional maximum scores depending on the kind of personality disorder, for example, 25: histrionic, 38: antisocial.

### Analyses

Data processing and statistical analyses were performed in MATLAB (2010, The MathWorks, Inc., Natick, MA, USA) and IBM SPSS 27.0 and 29.0 including the bootstrapping tool (IBM Corp. 2020).

Following the requirements of Field (2018), a normal distribution of the residuals was examined for all regression models. This could not be confirmed for the schizoid and borderline PD models in the NP group (examined using Kolmogorov-Smirnov and Shapiro-Wilk tests, among others). In addition, we found heteroscedasticity of the residuals in the NP group for all PDs investigated. Therefore, correlational, analysis of variance, and linear regression analyses were performed with 1000 resamples bootstrapping with bias-corrected and accelerated confidence intervals (BCa CIs) to provide more robust statistics (Field, 2018).

### Models

**Figure 1.**
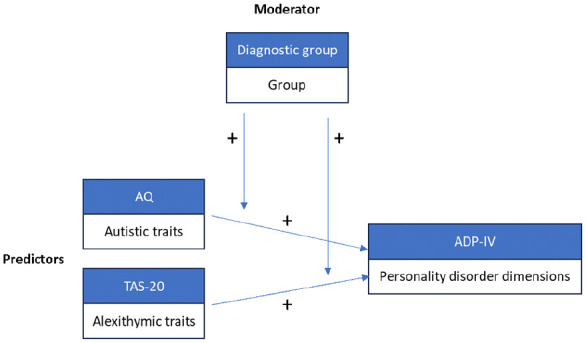
Model depicting the positive associations of autistic and alexithymic traits on personality disorder dimensions with group as the moderating variable.

We tested whether autistic, alexithymic, or both traits explained variance of PD dimensions (Figure 1). To follow the idea of a dimensional construct, we conducted multiple regression analyses in the total sample first. The four selected PD dimensions, namely schizoid, borderline, avoidant, and OCPD, served as dependent variables. AQ and TAS-20 scores served as independent variables of main interest. In addition, we examined the effect of diagnostic group (AP, NAP, NP) as a moderator between autistic and alexithymic traits and PD dimensions (Figure 1). For this approach, we created dummy variables for the three diagnostic groups (Field, 2018). We then created an interaction term between the predictors and the moderators with NP as the reference group. Next, we included the diagnostic groups, as well as the interaction terms of diagnostic groups and AQ and TAS-20 scores, respectively.

We entered depressive and socially anxious symptoms as covariates to adjust for them because both symptoms have been shown to correlate with AQ and TAS-20 scores in autistic and non-autistic individuals in previous studies (e.g. Bejerot et al., 2014; Bloch et al., 2021; Carpita et al., 2024; Spain et al., 2018; van Heijst et al., 2020). Age was also added as a covariate. To avoid sample-specific sampling errors, which could potentially lead to problems with generalization (Smith, 2018), we decided to include predictors and covariates simultaneously. Due to evidence of sex-based biases in both the TAS (Levant et al., 2022) and the AQ (Belcher et al., 2023), particularly at the scalar level (i.e. lack of invariance at the level of item intercepts), we repeated the analyses for each sex separately (Supplemental Tables S3a–S6b).

To identify associations among the variables, simple correlation analyses were run for each group (Supplemental Tables S2a–S2c). Multicollinearity of predictor variables was checked for by computing the variance inflation factor (VIF) and the tolerance statistic for each analysis. Values of VIF and of tolerance were below cutoff criteria in all analyses, suggesting that multicollinearity was negligible. Although tests did not indicate significant multicollinearity, moderate correlations between TAS-20 and AQ scores were found in each group with Pearson’s *r* below 0.8 (Supplemental Tables S2a–S2c).

## Results

### Sample characterization

Alexithymia, defined as TAS-20 scores ⩾61 (Bagby et al., 1994) was found in 58.4% of AP and 49.0% of NAP, while only 6.0% of NP had scores suggestive of alexithymia. AP and NAP showed similar frequency rates of co-occurring psychiatric conditions (Supplemental Table S1). In both groups, depression was the most common co-occurring psychiatric condition (AP: 58.4% and NAP: 49.0%, respectively), followed by social anxiety (AP: 23.6% and NAP: 25.5%, respectively).

The most prevalent three PD dimensions according to ADP-IV in AP were OCPD (77.5%), avoidant (71.9%), and paranoid (46.1%) PD dimensions. Although schizoid PD dimension was only found in 23% of AP in our study, we decided to investigate schizoid PD in the context of autism and alexithymia due to previous results, which found schizoid PD as the most common PD in their autistic samples (Lugnegård et al., 2012; Strunz et al., 2015). In NAP, the three most prevalent PD dimensions were OCPD (80.4%), avoidant (76.5%), and narcissistic (70.6%) PD dimensions. In NP, OCPD (33.3%), narcissistic (23.8%), paranoid and antisocial (17.9%) PD dimensions were the most common PD dimensions. A more detailed distribution of ADP-IV scores can be found in Supplemental Figure S1.

### Regression models

To address our hypothesis that variance in PD dimensions can be explained by both autistic and alexithymic traits, we entered the covariates, autistic and alexithymic traits of the total sample first. To consider potential group-related effects on the PD dimension, we then added group and the interaction effect of group and autistic and alexithymic traits, respectively (Tables 3to 6). Results affecting the covariates (e.g. age, symptoms of depression, and social anxiety) and the sex-specific subsamples are summarized in tables (Tables 3–6 and Supplemental Tables S3a–S6b, respectively).

**Table 3.** Linear models of predictors of schizoid PD dimension.

Models	Predictors	*b*	*SE B*	*β*	*p*	BCa 95% confidence interval	
						Lower	Upper
1	Constant	3.67	1.99		.072	0.01	7.47
	Age	0.09	0.04	.12	.017*	0.02	0.18
	BDI-II	0.14	0.05	.19	.004**	0.06	0.22
	LSAS	0.05	0.02	.19	.013*	0.01	0.09
	AQ	0.20	0.05	.29	<.001***	0.10	0.30
	TAS-20	0.09	0.04	.14	.046*	0.00	0.17
2	Constant	−1.13	3.61		.757	−8.35	5.86
	Age	0.08	0.04	.10	.042*	0.00	0.16
	BDI-II	0.09	0.05	.13	.043*	0.01	0.17
	LSAS	0.05	0.02	.19	.009**	0.02	0.09
	AQ	0.29	0.13	.42	.036*	0.03	0.53
	TAS-20	0.17	0.06	.27	.003**	0.04	0.28
	AP	7.91	5.60	.45	.168	−1.85	18.41
	NAP	18.81	6.29	.92	<.001***	6.99	31.24
	AP × AQ	−0.10	0.16	−.22	.535	−0.45	0.28
	NAP × AQ	−0.22	0.16	−.36	.178	−0.57	0.18
	AP × TAS-20	−0.11	0.11	−.41	.300	−0.34	0.07
	NAP × TAS-20	−0.19	0.11	−.57	.068	−0.39	0.01

AP: autistic participants; NAP: non-autistic participants with difficulties in social interaction. Neurotypical participants were used as reference group. 95% bias-corrected and accelerated confidence intervals. Confidence intervals and standard errors based on 1000 bootstrap samples.

* *p* < .05. **p < .01. ***p < .001.

**Table 4.** Linear models of predictors of borderline PD dimension.

Models	Predictors	*b*	*SE B*	*β*	*p*	BCa 95% confidence interval	
						Lower	Upper
1	Constant	16.50	2.66		<.001***	11.44	21.49
	Age	−0.15	0.06	−.15	.007**	−0.27	−0.04
	BDI-II	0.50	0.07	.53	<.001***	0.38	0.62
	LSAS	0.02	0.03	.04	.630	−0.04	0.07
	AP	−0.05	0.10	−.06	.585	−0.24	0.13
	TAS-20	0.19	0.07	.23	.010*	0.05	0.34
2	Constant	9.43	4.95		.053	0.02	20.01
	Age	−0.13	0.06	−.12	.034*	−0.23	−0.02
	BDI-II	0.44	0.07	.47	<.001***	0.29	0.59
	LSAS	0.04	0.03	.11	.186	−0.02	0.09
	AQ	0.10	0.21	.11	.618	−0.35	0.43
	TAS-20	0.28	0.10	.35	.006**	0.08	0.50
	AP	1.54	7.40	.07	.841	−11.51	13.90
	NAP	6.81	8.53	.26	.420	−9.76	24.57
	AP × AQ	−0.15	0.25	−.25	.575	−0.65	0.45
	NAP × AQ	0.10	0.28	.12	.716	−0.45	0.73
	AP × TAS-20	−0.08	0.15	−.22	.625	−0.36	0.25
	NAP × TAS-20	−0.19	0.20	−.44	.307	−0.59	0.17

AP: autistic participants; NAP: non-autistic participants with difficulties in social interaction. Neurotypical participants were used as reference group. 95% bias-corrected and accelerated confidence intervals. Confidence intervals and standard errors based on 1000 bootstrap samples.

* *p* < .05. **p < .01. ***p < .001.

**Table 5.** Linear models of predictors of avoidant PD dimension.

Models	Predictors	*b*	*SE B*	*β*	*p*	BCa 95% confidence interval	
						Lower	Upper
1	Constant	3.98	1.89		.034*	0.26	7.63
	Age	0.01	0.04	.01	.893	−0.09	0.10
	BDI-II	0.20	0.06	.22	.002**	0.09	0.31
	LSAS	0.14	0.02	.42	<.001***	0.10	0.19
	AQ	0.17	0.06	.19	.005**	0.06	0.28
	TAS-20	0.12	0.05	.15	.014*	0.03	0.22
2	Constant	1.11	4.24		.807	−6.95	9.57
	Age	0.01	0.05	.01	.879	−0.09	0.11
	BDI-II	0.16	0.06	.17	.007**	0.05	0.27
	LSAS	0.14	0.02	.43	<.001***	0.10	0.19
	AQ	0.27	0.14	.31	.056	0.00	0.50
	TAS-20	0.15	0.09	.18	.118	−0.04	0.33
	AP	9.62	6.38	.43	.137	−2.65	22.73
	NAP	5.34	6.69	.20	.426	−8.46	16.72
	AP × AQ	−0.26	0.19	−.45	.166	−0.66	0.19
	NAP × AQ	−0.11	0.17	−.15	.499	−0.45	0.35
	AP × TAS-20	−0.03	0.12	−.10	.771	−0.27	0.18
	NAP × TAS-20	−0.02	0.12	−.04	.865	−0.25	0.20

AP: autistic participants; NAP: non-autistic participants with difficulties in social interaction.

Neurotypical participants were used as reference group. 95% bias-corrected and accelerated confidence intervals. Confidence intervals and standard errors based on 1000 bootstrap samples.

* *p* < .05. **p < .01. ***p < .001.

**Table 6.** Linear models of predictors of OCPD dimension.

Models	Predictors	*b*	*SE B*	*β*	*p*	BCa 95% confidence interval	
						Lower	Upper
1	Constant	14.54	2.24		<.001***	10.16	18.71
	Age	0.01	0.05	.01	.881	−0.09	0.10
	BDI-II	0.13	0.05	.16	.009**	0.03	0.23
	LSAS	0.08	0.02	.27	.004**	0.04	0.13
	AQ	0.27	0.06	.34	<.001***	0.16	0.37
	TAS-20	0.01	0.05	.02	.790	−0.09	0.12
2	Constant	13.54	4.88		.004**	4.34	24.00
	Age	0.02	0.05	.02	.735	−0.08	0.11
	BDI-II	0.14	0.06	.17	.011*	0.03	0.24
	LSAS	0.09	0.02	.30	.003**	0.05	0.14
	AQ	0.51	0.16	.65	.002**	0.23	0.78
	TAS-20	−0.04	0.09	−.06	.638	−0.21	0.13
	AP	−5.57	6.64	−.28	.403	−18.29	6.74
	NAP	−5.34	7.70	−.23	.493	−20.78	8.48
	AP × AQ	−0.17	0.20	−.32	.424	−0.58	0.25
	NAP × AQ	−0.26	0.21	−.39	.207	−0.73	0.13
	AP × TAS-20	0.09	0.12	.28	.479	−0.17	0.32
	NAP × TAS-20	0.16	0.15	.41	.294	−0.13	0.48

AP: autistic participants; NAP: non-autistic participants with difficulties in social interaction.

Neurotypical participants were used as a reference group. 95% bias-corrected and accelerated confidence intervals. Confidence intervals and standard errors based on 1000 bootstrap samples.

* *p* < .05. **p < .01. ***p < .001.

### Schizoid PD dimension

The final model including autistic and alexithymic traits as predictors; group as a moderator; and age, depressive symptoms, and socially anxious symptoms as covariates explained 58.9% (adjusted *R*^2^ = 56.8%) of variance of schizoid PD dimension (*F*(11, 211) = 27.53; *p* < .001, *f*^2^ = 0.28). Autistic and alexithymic traits were both significant predictors of schizoid PD dimension. Relative to NP, schizoid dimension was significantly increased in NAP (Table 3).

### Borderline PD dimension

The final model explained 47.6% (adjusted *R*^2^ = 44.9%) of variance (*F*(11, 211) = 17.42; *p* < .001, *f*^2^ = 0.09) with alexithymic traits being significant predictors of borderline PD dimension (Table 4).

### Avoidant PD dimension

The final model explained 72.4% (adjusted *R*^2^ = 71.0%) of variance (*F*(11, 211) = 50.36; *p* < .001, *f*^2^ = 1.21) with neither autistic nor alexithymic traits being significant predictors of avoidant PD dimension (Table 5).

### OCPD dimension

The final model explained 49.5% (adjusted *R*^2^ = 46.9%) of variance (*F*(11, 211) = 18.84; *p* < .001, *f*^2^ = 0.11), with autistic traits being significant predictors of OCPD dimension (Table 6).

## Discussion

The present study examined the association of autistic and alexithymic traits with PD dimensions in adults with a confirmed diagnosis of autism, adults with social interaction difficulties due to a psychiatric condition other than autism, and adults without any psychological complaints. Considering autistic, alexithymic, and PD dimensions as continua, analyses were performed in the total sample first, while controlling for age, depressive symptoms, and socially anxious symptoms. To investigate the potential effect of the underlying core condition on autistic and alexithymic trait severity and thus on the PD dimensions, we added the diagnostic group as the moderator to the analyses.

The prevalence rates of alexithymia were in line with previous reports of alexithymic traits in equivalent samples for each diagnostic group (AP: Berthoz & Hill, 2005; Kinnaird et al., 2019; Milosavljevic et al., 2016; Morie et al., 2019; NAP: Cox et al., 1995; Leweke et al., 2012; NP: Fietz et al., 2018). Furthermore, the prevalence rates of depression and social anxiety in the autistic group are consistent with other studies about autism in adulthood (Albantakis et al., 2018; Hedley et al., 2018; Maddox & White, 2015; Spain et al., 2016). Thus, our study sample realistically met the characteristics of autistic adults including co-occurring psychiatric conditions. Importantly, AP and NAP showed similar rates of co-occurring psychiatric conditions, leaving the underlying core condition as the main difference.

Our hypothesis that both autistic and alexithymic traits predict PD dimension was confirmed for schizoid PD dimension. NAP group was also a significant predictor of schizoid PD dimension. These findings add to previous research by Booules-Katri et al. (2019), who found socio-cognitive impairments in autistic individuals and patients with schizotypal-schizoid PD, indicating a symptomatic overlap between autism and schizoid PD. Regarding alexithymic traits, Garofalo and colleagues (2018) found that reduced emotional awareness predicted schizoid PD in a non-clinical sample. Thus, our results complement previous findings and emphasize the relevance of both autistic and alexithymic traits when investigating schizoid PD dimension.

Autistic traits also predicted the OCPD dimension. This is in line with a previous study, which found a highly significant correlation (*p* < .001) between AQ scores and OCPD traits (Gadelkarim et al., 2019). The positive association between autistic traits and OCPD dimension is also reflected in overlapping diagnostic criteria of autism and OCPD, such as interest in symmetries, rule-based preference, and other stereotypes (Lai & Baron-Cohen, 2015). In the context of emotion regulation, autistic individuals, patients with obsessive compulsive disorder (OCD), and persons with high levels of alexithymia frequently apply “suppression,” defined as the attempt to hide, inhibit, or reduce ongoing emotion-expressive behavior, as a maladaptive strategy (Cutuli, 2014; Ferreira et al., 2021; Gross & John, 2003; Gross & Levenson, 1993; Preece et al., 2023). However, most research suggesting alexithymia to mediate OCD symptoms did not control for autistic traits (e.g. Eddy & Hansen, 2021; Pozza et al., 2015; Robinson & Freeston, 2014). In this regard, our findings extend the previous literature by investigating the relevance of both autistic and alexithymic traits on the OCPD dimension, while emphasizing the predictive value of autistic traits on the OCPD dimension.

Neither autistic nor alexithymic traits were significant predictors of the avoidant PD dimension, after adding diagnostic group as a moderator to the analysis. Instead, depressive and social anxious symptoms were significant predictors. Thus, we assume that additional characteristics of AP and NAP, such as depression and social anxiety as co-occurring psychiatric conditions, might have contributed to the relevance of depressive and socially anxious symptoms in the context of avoidance PD dimension. Social avoidance has been discussed as a hallmark of schizophrenia, autism, and PDs (Simon et al., 2021). A recent study (Beaurenaut et al., 2024) investigated avoidance learning in social contexts in autism. The researchers found correlations between anxiety-depression severity and reduced learning to engage with others across autistic and neurotypical participants. Considering avoidant PD as the chronic state of avoidance in social contexts (Fariba et al., 2024c), our results are in line with the findings by Beaurenaut et al. From a dimensional perspective, putting diagnostic group as a moderator aside, both autistic and alexithymic traits predicted avoidant PD dimension in our study. Loas and colleagues (2015) found avoidant PD and depression to be independent predictors of TAS-20 scores, while Li and colleagues (2023) found autistic traits to predict social avoidance and distress via perceived stress and interpersonal alienation in non-autistic people. Taken together, these findings indicate a complex interplay of depression, social anxiety, autism, and alexithymia in the context of avoidance.

Previous research found elevated AQ scores in participants with the diagnosis of borderline PD dimensions, suggesting an overlap between autism and borderline PD dimension (Dudas et al., 2017; Nanchen et al., 2016). In a non-clinical sample with young adults, participants with increased levels of both autistic and borderline traits described higher levels of suicidal ideation than the participants with increased borderline traits alone, despite similar levels of depressive symptoms (Chabrol & Raynal, 2018). Neurocognitive impairments in empathy, mentalization, and theory of mind as well as a shared vulnerability toward traumatic events have been suggested as other potential overlaps between borderline PD and autism more recently (Dell’ Osso et al., 2023; Vegni et al., 2021). Our results indicate that alexithymic rather than autistic traits are linked to the borderline PD dimension. As mentioned earlier, alexithymia may be interpreted as being a consequence of previously unelaborated traumatic experiences (Franzoni et al., 2013). Thus, it is important to explore potential traumatic experiences in the diagnostic assessment of disorders of social interaction. Given the high percentage of co-occurring alexithymia in autistic individuals (Kinnaird et al., 2019) and borderline PD (Kılıç et al., 2020), it is also essential to include alexithymia as a potential influencing factor, when investigating symptomatic overlaps between autism and borderline PD. This aspect appears to have been neglected in previous studies.

### Strengths and limitations

A strength of our study lies in the relatively large number of study participants. We included 224 study participants in total (AP: *n* = 89, NAP: *n* = 51, NP: *n* = 84), providing the robust statistical power of the analyses. Furthermore, we applied a multi-sample approach to examine the impact of the underlying core condition on the variables of interest because the origin of the samples (clinical or non-clinical data) matters when investigating psychiatric conditions (Aldao et al., 2010). The autistic sample in our study realistically met the characteristics of autistic adults, while the prevalence rates of alexithymia in the non-autistic clinical sample and the neurotypical sample in our study matched previous results from equivalent samples.

The statistical analyses chosen for this study do not indicate causality but correlation. As expected, we found high correlations among our variables of interest (Supplemental Tables S2a–S2c). However, tests performed indicated that multicollinearity was negligible.

A general limitation lies within potential measurement issues when assessing alexithymia and autistic traits across autistic and non-autistic individuals by applying psychometric tools like the TAS-20 and AQ (e.g. Williams & Gotham, 2021). While psychometric issues may influence the current results, this can only be further investigated once valid and reliable measures are available. We ran additional analyses for each sex separately (Supplemental Material) but did not include sex as a moderator into the analyses due to the number of covariates and analyses in relation to the participants included. Future studies should integrate sex as a potential confounding variable.

### Conclusion

Our study demonstrates that autistic traits were significant predictors of the OCPD dimension, while both autistic and alexithymic traits were significant predictors of the schizoid PD dimension. Furthermore, we found alexithymic traits to predict the borderline PD dimension. Taken together, our results point toward overlap between autistic traits and specific PD dimensions that are well recognized as differential diagnoses of autism, while alexithymia was differentially linked to the borderline PD dimension.

## Supplemental Material

sj-docx-1-aut-10.1177_13623613251338650 – Supplemental material for Alexithymic and autistic traits differentially predict personality disorder dimensions Supplemental material, sj-docx-1-aut-10.1177_13623613251338650 for Alexithymic and autistic traits differentially predict personality disorder dimensions by Laura Albantakis, Leonie Weindel, Marie-Luise Brandi, Imme C. Zillekens, Lara Henco, Hanna Thaler, Lena Schliephake and Leonhard Schilbach in Autism
