# Supplementary material for: Alexithymic and autistic traits differentially predict personality disorder dimensions
Source: Autism. 2025 Jun 1;29(10):2463–76. doi: 10.1177/13623613251338650 (PMC12417610; doi:10.1177/13623613251338650)
Supplement: sj-docx-1-aut-10.1177_13623613251338650 – Supplemental material for Alexithymic and autistic traits differentially predict personality disorder dimensions [file sj-docx-1-aut-10.1177_13623613251338650.docx]

**Supplementary Material**

**Table S1**

*Co-occurring psychiatric conditions*

| **Conditions^1^** | **AP** | **NAP** |
| --- | --- | --- |
| Depression | 52 (58.4) | 25 (49.0) |
| Social Anxiety | 21 (23.6) | 13 (25.5) |
| Attention deficit hyperactivity disorder (ADHD) | 7 (7.9) | 5 (9.8) |
| Obsessive-compulsive disorder (OCD) | 4 (4.5) | 3 (5.9) |
| No psychiatric conditions | 19 (21.3) | 12 (23.5) |
| ^1^According to ICD-10 criteria in N (%), at least suspected diagnosis. Multiple diagnoses are possible.  AP: Autistic participants; NAP: non-autistic participants. | | |

**Figure S1**

*Boxplots of PD dimensions in all three diagnostic groups*

**
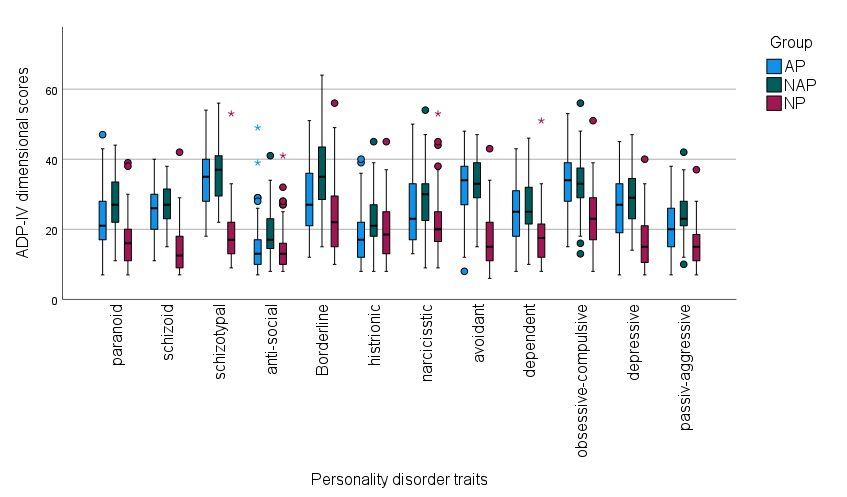
**

*Note.* AP: Autistic participants; NAP: non-autistic participants; NP: neurotypical participants.

**Table S2a**

*Correlations of study variables in AP*

|  | | **Age** | **Sex** | **BDI-II** | **LSAS** | **AQ** | **TAS-20** |
| --- | --- | --- | --- | --- | --- | --- | --- |
| **Age** | *r* | 1.00 | 0.15 | 0.12 | 0.22 | 0.30 | 0.24 |
|  | *p* |  | .074 | .123 | .021* | .002** | .011* |
|  | 95% BCa-KI |  | [-0.04, 0.37] | [-0.09, 0.33] | [0.01, 0.40] | [0.06, 0.51] | [0.02, 0.47] |
| **Sex** | *r* | 0.15 | 1.00 | 0.09 | 0.21 | 0.15 | -0.01 |
|  | *p* | .074 |  | .194 | .023* | .084 | .465 |
|  | 95% BCa-KI | [-0.04, 0.37] |  | [-0.14, 0.31] | [0.03, 0.38] | [-0.08, 0.34] | [-0.21, 0.19] |
| **BDI-II** | *r* | 0.12 | 0.09 | 1.00 | 0.41 | 0.16 | 0.34 |
|  | *p* | .123 | .194 |  | <.001*** | .064 | .001** |
|  | 95% BCa-KI | [-0.09, 0.33] | [-0.14, 0.31] |  | [0.22, 0.56] | [-0.08, 0.37] | [0.14, 0.51] |
| **LSAS** | *r* | 0.22 | 0.21 | 0.41 | 1.00 | 0.46 | 0.33 |
|  | *p* | .021* | .023* | <.001*** |  | <.001*** | .001** |
|  | 95% BCa-KI | [0.01, 0.40] | [0.03, 0.38] | [0.22, 0.56] |  | [0.31, 0.59] | [0.12, 0.52] |
| **AQ** | *r* | 0.30 | 0.15 | 0.16 | 0.46 | 1.00 | 0.52 |
|  | *p* | .002** | .084 | .064 | <.001*** |  | <.001*** |
|  | 95% BCa-KI | [0.06, 0.51] | [-0.08, 0.34] | [-0.08, 0.37] | [0.31, 0.59] |  | [0.33, 0.69] |
| **TAS-20** | *r* | 0.24 | -0.01 | 0.34 | 0.33 | 0.52 | 1.00 |
|  | *p* | .011* | .465 | .001** | .001** | <.001*** |  |
|  | 95% BCa-KI | [0.02, 0.47] | [-0.21, 0.19] | [0.14, 0.51] | [0.12, 0.52] | [0.33, 0.69] |  |
| **ADP-IV**  **Schizoid** | *r* | 0.22 | -0.18 | 0.25 | 0.42 | 0.42 | 0.35 |
|  | *p* | .022* | .048* | .009** | <.001*** | <.001*** | <.001*** |
|  | 95% BCa-KI | [0.01, 0.39] | [-0.36, 0.02] | [0.06, 0.42] | [0.21, 0.60] | [0.25, 0.59] | [0.13, 0.54] |
| **ADP-IV Borderline** | *r* | -0.02 | -0.05 | 0.58 | 0.34 | 0.16 | 0.37 |
|  | *p* | .427 | .328 | <.001*** | <.001*** | .064 | <.001*** |
|  | 95% BCa-KI | [-0.27, 0.25] | [-0.23, 0.16] | [0.42, 0.72] | [0.15, 0.50] | [-0.06, 0.37] | [0.15, 0.55] |
| **ADP-IV**  **Avoidant** | *r* | 0.10 | 0.05 | 0.48 | 0.65 | 0.33 | 0.37 |
|  | *p* | .166 | .330 | <.001*** | <.001*** | .001** | <.001*** |
|  | 95% BCa-KI | [-0.11, 0.30] | [-0.18, 0.25] | [0.26, 0.66] | [0.50, 0.77] | [0.12, 0.52] | [0.15, 0.55] |
| **ADP-IV**  **OC** | *r* | 0.17 | 0.09 | 0.30 | 0.56 | 0.49 | 0.37 |
|  | *p* | .051 | .212 | .002** | <.001*** | <.001*** | <.001*** |
|  | 95% BCa-KI | [-0.05, 0.39] | [-0.12, 0.32] | [0.07, 0.48] | [0.37, 0.70] | [0.29, 0.64] | [0.15, 0.56] |
| *Indicates *p*<.05. **Indicates *p*<.01. ***Indicates *p*<.001.  Results are based on 1,000 bootstrap samples (95% BCa-confident intervals, bias corrected and accelerated). | | | | | | | |

**Table S2b**

*Correlations of study variables in NAP*

|  | | **Age** | **Sex** | **BDI-II** | **LSAS** | **AQ** | **TAS-20** |
| --- | --- | --- | --- | --- | --- | --- | --- |
| **Age** | *r* | 1.00 | -0.09 | -0.06 | -0.16 | -0.12 | 0.00 |
|  | *p* |  | .256 | .347 | .132 | .205 | .492 |
|  | 95% BCa-KI |  | [-0.38, 0.21] | [-0.33, 0.20] | [-0.40, 0.11] | [-0.45, 0.21] | [-0.27, 0.28] |
| **Sex** | *r* | -0.09 | 1.00 | 0.01 | 0.18 | 0.06 | -0.01 |
|  | *p* | .256 |  | .478 | .106 | .346 | .462 |
|  | 95% BCa-KI | [-0.38, 0.21] |  | [-0.28, 0.31] | [-0.08, 0.44] | [-0.25, 0.37] | [-0.30, 0.27] |
| **BDI-II** | *r* | -0.06 | 0.01 | 1.00 | 0.13 | 0.12 | 0.25 |
|  | *p* | .347 | .478 |  | .184 | .198 | .040* |
|  | 95% BCa-KI | [-0.33, 0.20] | [-0.28, 0.31] |  | [-0.14, 0.38] | [-0.21, 0.41] | [-0.05, 0.50] |
| **LSAS** | *r* | -0.16 | 0.18 | 0.13 | 1.00 | 0.36 | 0.51 |
|  | *p* | .132 | .106 | .184 |  | .005** | <.001*** |
|  | 95% BCa-KI | [-0.40, 0.11] | [-0.08, 0.44] | [-0.14, 0.38] |  | [0.15, 0.56] | [0.27, 0.69] |
| **AQ** | *r* | -0.12 | 0.06 | 0.12 | 0.36 | 1.00 | 0.43 |
|  | *p* | .205 | .346 | .198 | .005** |  | .001** |
|  | 95% BCa-KI | [-0.45, 0.21] | [-0.25, 0.37] | [-0.21, 0.41] | [0.15, 0.56] |  | [0.15, 0.64] |
| **TAS-20** | *r* | 0.00 | -0.01 | 0.25 | 0.51 | 0.43 | 1.00 |
|  | *p* | .492 | .462 | .040* | <.001*** | .001** |  |
|  | 95% BCa-KI | [-0.27, 0.28] | [-0.30, 0.27] | [-0.05, 0.50] | [0.27, 0.69] | [0.15, 0.64] |  |
| **ADP-IV Schizoid** | *r* | 0.08 | -0.10 | 0.32 | 0.20 | 0.18 | 0.16 |
|  | *p* | .292 | .242 | .010* | .084 | .109 | .125 |
|  | 95% BCa-KI | [-0.18, 0.33] | [-0.38, 0.22] | [0.05, 0.57] | [-0.05, 0.45] | [-0.15, 0.46] | [-0.10, 0.41] |
| **ADP-IV**  **Borderline** | *r* | -0.25 | 0.11 | 0.40 | 0.24 | 0.30 | 0.31 |
|  | *p* | .037* | .222 | .002** | .049* | .015* | .012* |
|  | 95% BCa-KI | [-0.48, 0.01] | [-0.22, 0.41] | [0.09, 0.64] | [-0.04, 0.47] | [0.02, 0.55] | [0.01, 0.59] |
| **ADP-IV Avoidant** | *r* | -0.10 | -0.01 | 0.23 | 0.58 | 0.48 | 0.59 |
|  | *p* | .247 | .464 | .050 | <.001*** | <.001*** | <.001*** |
|  | 95% BCa-KI | [-0.36, 0.17] | [-0.29, 0.25] | [-0.04, 0.50] | [0.31, 0.80] | [0.31, 0.64] | [0.40, 0.76] |
| **ADP-IV**  **OC** | *r* | 0.01 | 0.10 | 0.34 | 0.38 | 0.46 | 0.47 |
|  | *p* | .473 | .241 | .008** | .003** | <.001*** | <.001*** |
|  | 95% BCa-KI | [-0.29, 0.32] | [-0.18, 0.41] | [0.04, 0.62] | [0.16, 0.57] | [0.19, 0.67] | [0.22, 0.66] |
| *Indicates *p*<.05. **Indicates *p*<.01. ***Indicates *p*<.001.  Results are based on 1,000 bootstrap samples (95% BCa-confident intervals, bias corrected and accelerated). | | | | | | | |

**Table S2c**

*Correlations of study variables in NP*

|  | | | | **Age** | | **Sex** | | **BDI-II** | | **LSAS** | | **AQ** | **TAS-20** |
| --- | --- | --- | --- | --- | --- | --- | --- | --- | --- | --- | --- | --- | --- |
| **Age** | *r* | | 1.00 | | -0.02 | | -0.05 | | -0.26 | | 0.02 | | -0.22 |
|  | *p* | |  | | .446 | | .339 | | .009** | | .448 | | .024* |
|  | 95% BCa-KI | |  | | [-0.23, 0.17] | | [-0.28, 0.24] | | [-0.45, -0.05] | | [-0.21, 0.24] | | [-0.45, 0.09] |
| **Sex** | *r* | | -0.02 | | 1.00 | | 0.03 | | 0.01 | | -0.32 | | -0.25 |
|  | *p* | | .446 | |  | | .413 | | .478 | | .002** | | .012* |
|  | 95% BCa-KI | | [-0.23, 0.17] | |  | | [-0.20, 0.23] | | [-0.27, 0.27] | | [-0.50, -0.14] | | [-0.43, -0.08] |
| **BDI-II** | *r* | | -0.05 | | 0.03 | | 1.00 | | 0.25 | | 0.31 | | 0.39 |
|  | *p* | | .339 | | .413 | |  | | .011* | | .002** | | <.001*** |
|  | 95% BCa-KI | | [-0.28, 0.24] | | [-0.20, 0.23] | |  | | [0.06, 0.44] | | [0.07, 0.52] | | [0.21, 0.59] |
| **LSAS** | *r* | | -0.26 | | 0.01 | | 0.25 | | 1.00 | | 0.26 | | 0.30 |
|  | *p* | | .009** | | .478 | | .011* | |  | | .008** | | .003** |
|  | 95% BCa-KI | | [-0.45, -0.05 | | [-0.27, 0.27] | | [0.06, 0.44] | |  | | [0.05, 0.45] | | [0.11, 0.45] |
| **AQ** | *r* | | 0.02 | | -0.32 | | 0.31 | | 0.26 | | 1.00 | | 0.29 |
|  | *p* | | .448 | | .002** | | .002** | | .008** | |  | | .004** |
|  | 95% BCa-KI | | [-0.21, 0.24] | | [-0.50, -0.14] | | [0.07, 0.52] | | [0.05, 0.45] | |  | | [0.08, 0.50] |
| **TAS-20** | *r* | | -0.22 | | -0.25 | | 0.39 | | 0.30 | | 0.29 | | 1.00 |
|  | *p* | | .024* | | .012* | | <.001*** | | .003** | | .004** | |  |
|  | 95% BCa-KI | | [-0.45, 0.09] | | [-0.43, -0.08] | | [0.21, 0.59] | | [0.11, 0.46] | | [0.08, 0.50] | |  |
| **ADP-IV Schizoid** | *r* | | 0.13 | | -0.18 | | 0.31 | | 0.33 | | 0.39 | | 0.39 |
|  | *p* | | .120 | | .050 | | .002** | | .001** | | <.001*** | | <.001*** |
|  | 95% BCa-KI | | [-0.11, 0.37] | | [-0.37, 0.03] | | [0.08, 0.52] | | [0.17, 0.49] | | [0.14, 0.58] | | [0.15, 0.58] |
| **ADP-IV Borderline** | *r* | | -0.09 | | 0.06 | | 0.60 | | 0.27 | | 0.23 | | 0.45 |
|  | *p* | | .223 | | .283 | | <.001*** | | .006** | | .017* | | <.001*** |
|  | 95% BCa-KI | | [-0.30, 0.18] | | [-0.13, 0.25] | | [0.44, 0.72] | | [0.08, 0.45] | | [-0.05, 0.48] | | [0.23, 0.63] |
| **ADP-IV**  **Avoidant** | *r* | | -0.01 | | -0.16 | | 0.41 | | 0.48 | | 0.38 | | 0.40 |
|  | *p* | | .477 | | .073 | | <.001*** | | <.001*** | | <.001*** | | <.001*** |
|  | 95% BCa-KI | | [-0.22, 0.25] | | [-0.36, 0.03] | | [0.17, 0.62] | | [0.30, 0.63] | | [0.14, 0.57] | | [0.14, 0.61] |
| **ADP-IV**  **OC** | *r* | | -0.06 | | -0.09 | | 0.33 | | 0.30 | | 0.39 | | 0.13 |
|  | | *p* | | .311 | | .209 | | .001** | | .003** | | <.001*** | .123 |
|  |  | 95% BCa-KI | | [-0.27, 0.18] | | [-0.30, 0.14] | | [0.12, 0.53] | | [0.10, 0.48] | | [0.14, 0.58] | [-0.16, 0.36] |
| *Indicates *p*<.05. **Indicates *p*<.01. ***Indicates *p*<.001.  Results are based on 1,000 bootstrap samples (95% BCa-confident intervals, bias corrected and accelerated). | | | | | | | | | | | | | |

**Table S3a**

*Linear models of predictors of schizoid PD dimension in males. 95 % bias corrected and accelerated confidence intervals. Confidence intervals and standard errors based on 1,000 bootstrap samples.*

| Models | Predictors | *b* | *SE B* | *β* | *p* | BCa 95%  Confidence interval | |
| --- | --- | --- | --- | --- | --- | --- | --- |
|  |  |  |  |  |  | Lower | Upper |
| 1 | (Constant) | 4.46 | 3.06 |  | .141 | -1.80 | 10.94 |
|  | Age | 0.05 | 0.05 | .07 | .262 | -0.04 | 0.15 |
|  | BDI-II | 0.16 | 0.06 | .22 | .009** | 0.05 | 0.27 |
|  | LSAS | 0.04 | 0.02 | .15 | .089 | -0.01 | 0.09 |
|  | AQ | 0.24 | 0.06 | .32 | <.001*** | 0.12 | 0.36 |
|  | TAS-20 | 0.10 | 0.06 | .15 | .101 | -0.03 | 0.22 |
| 2 | (Constant) | -9.97 | 7.42 |  | .174 | -22.86 | 6.06 |
|  | Age | 0.02 | 0.05 | .03 | .640 | -0.07 | 0.14 |
|  | BDI-II | 0.10 | 0.06 | .14 | .111 | -0.02 | 0.21 |
|  | LSAS | 0.04 | 0.02 | .17 | .056 | -0.01 | 0.09 |
|  | AQ | 0.38 | 0.20 | .51 | .054 | -0.04 | 0.67 |
|  | TAS-20 | 0.36 | 0.12 | .55 | .004** | 0.12 | 0.58 |
|  | AP | 17.22 | 9.31 | 1.06 | .060 | -1.89 | 34.63 |
|  | NAP | 28.25 | 10.05 | 1.49 | .004** | 8.53 | 47.68 |
|  | AP x AQ | -0.13 | 0.22 | -.30 | .532 | -0.54 | 0.40 |
|  | NAP x AQ | -0.26 | 0.23 | -.46 | .239 | -0.70 | 0.27 |
|  | AP x TAS-20 | -0.29 | 0.16 | -1.17 | .049* | -0.59 | 0.02 |
|  | NAP x TAS-20 | -0.37 | 0.17 | -1.19 | .015* | -0.69 | -0.04 |

AP: Autistic participants; NAP: Non-autistic participants with difficulties in social interaction.

Neurotypical participants were used as reference group.

*Indicates *p* < .05. **Indicates *p* < .01. ***Indicates *p* < .001.

**Table S3b**

*Linear models of predictors of schizoid PD dimension in females. 95 % bias corrected and accelerated confidence intervals. Confidence intervals and standard errors based on 1,000 bootstrap samples.*

| Models | Predictors | *b* | *SE B* | *β* | *P* | BCa 95%  Confidence interval | |
| --- | --- | --- | --- | --- | --- | --- | --- |
|  |  |  |  |  |  | Lower | Upper |
| 1 | (Constant) | 3.201 | 2.71 |  | .242 | -1.76 | 8.01 |
|  | Age | 0.155 | 0.06 | .19 | .012* | 0.03 | 0.30 |
|  | BDI-II | 0.093 | 0.07 | .14 | .172 | -0.03 | 0.21 |
|  | LSAS | 0.098 | 0.03 | .39 | <.001*** | 0.03 | 0.17 |
|  | AQ | 0.082 | 0.08 | .13 | .325 | -0.08 | 0.22 |
|  | TAS-20 | 0.05 | 0.06 | .09 | .427 | -0.09 | 0.19 |
| 2 | (Constant) | -0.538 | 3.99 |  | .882 | -8.51 | 6.33 |
|  | Age | 0.188 | 0.07 | .23 | .009** | 0.06 | 0.35 |
|  | BDI-II | 0.082 | 0.07 | .12 | .246 | -0.06 | 0.23 |
|  | LSAS | 0.092 | 0.03 | .37 | .008** | 0.03 | 0.16 |
|  | AQ | 0.139 | 0.17 | .23 | .415 | -0.25 | 0.36 |
|  | TAS-20 | 0.102 | 0.07 | .18 | .168 | -0.04 | 0.29 |
|  | AP | -0.613 | 8.21 | -.03 | .943 | -15.97 | 13.32 |
|  | NAP | 15.756 | 11.83 | .78 | .162 | -9.17 | 44.98 |
|  | AP x AQ | 0.223 | 0.31 | .47 | .434 | -0.32 | 1.42 |
|  | NAP x AQ | -0.122 | 0.25 | -.21 | .594 | -0.61 | 0.52 |
|  | AP x TAS-20 | -0.187 | 0.21 | -.64 | .361 | -0.59 | 0.07 |
|  | NAP x TAS-20 | -0.181 | 0.20 | -.54 | .316 | -0.55 | 0.13 |

AP: Autistic participants; NAP: Non-autistic participants with difficulties in social interaction.

Neurotypical participants were used as reference group.

*Indicates *p* < .05. **Indicates *p* < .01. ***Indicates *p* < .001.

**Table S4a**

*Linear models of predictors of borderline PD dimension in males. 95 % bias corrected and accelerated confidence intervals. Confidence intervals and standard errors based on 1,000 bootstrap samples.*

| Models | Predictors | *b* | *SE B* | *β* | *P* | BCa 95%  Confidence interval | |
| --- | --- | --- | --- | --- | --- | --- | --- |
|  |  |  |  |  |  | Lower | Upper |
| 1 | (Constant) | 13.91 | 4.30 |  | .003** | 6.17 | 23.16 |
|  | Age | -0.09 | 0.08 | -.09 | .262 | -0.24 | 0.06 |
|  | BDI-II | 0.47 | 0.08 | .48 | <.001*** | 0.32 | 0.64 |
|  | LSAS | 0.06 | 0.04 | .16 | .149 | -0.02 | 0.13 |
|  | AQ | -0.09 | 0.11 | -.08 | .419 | -0.27 | 0.11 |
|  | TAS-20 | 0.17 | 0.11 | .19 | .100 | -0.04 | 0.39 |
| 2 | (Constant) | -7.94 | 8.78 |  | .384 | -24.05 | 12.78 |
|  | Age | -0.10 | 0.09 | -.10 | .239 | -0.27 | 0.06 |
|  | BDI-II | 0.39 | 0.10 | .40 | <.001*** | 0.19 | 0.60 |
|  | LSAS | 0.08 | 0.04 | .23 | .025* | 0.01 | 0.14 |
|  | AQ | 0.57 | 0.29 | .56 | .035* | 0.01 | 0.92 |
|  | TAS-20 | 0.41 | 0.17 | .46 | .006** | 0.12 | 0.69 |
|  | AP | 18.79 | 11.76 | .85 | .117 | -4.00 | 37.71 |
|  | NAP | 34.15 | 12.89 | 1.32 | .005** | 9.16 | 58.13 |
|  | AP x AQ | -0.69 | 0.34 | -1.17 | .045* | -1.36 | 0.19 |
|  | NAP x AQ | -0.67 | 0.36 | -.86 | .062 | -1.40 | 0.28 |
|  | AP x TAS-20 | -0.20 | 0.22 | -.59 | .343 | -0.66 | 0.29 |
|  | NAP x TAS-20 | -0.38 | 0.25 | -.90 | .118 | -0.88 | 0.06 |

AP: Autistic participants; NAP: Non-autistic participants with difficulties in social interaction.

Neurotypical participants were used as reference group.

*Indicates *p* < .05. **Indicates *p* < .01. ***Indicates *p* < .001.

**Table S4b**

*Linear models of predictors of borderline PD dimension in females. 95 % bias corrected and accelerated confidence intervals. Confidence intervals and standard errors based on 1,000 bootstrap samples.*

| Models | Predictors | *b* | *SE B* | *β* | *p* | BCa 95%  Confidence interval | |
| --- | --- | --- | --- | --- | --- | --- | --- |
|  |  |  |  |  |  | Lower | Upper |
| 1 | (Constant) | 21.14 | 4.148 |  | <.001*** | 13.66 | 28.51 |
|  | Age | -0.27 | 0.1 | -.24 | .015* | -0.50 | -0.05 |
|  | BDI-II | 0.63 | 0.101 | .68 | <.001*** | 0.41 | 0.83 |
|  | LSAS | -0.09 | 0.046 | -.25 | .077 | -0.17 | 0.01 |
|  | AQ | 0.11 | 0.174 | .13 | .548 | -0.23 | 0.38 |
|  | TAS-20 | 0.18 | 0.105 | .23 | .112 | -0.04 | 0.41 |
| 2 | (Constant) | 16.50 | 6.225 |  | .012* | 4.19 | 28.40 |
|  | Age | -0.17 | 0.085 | -.16 | .039* | -0.35 | 0.01 |
|  | BDI-II | 0.56 | 0.094 | .61 | <.001*** | 0.35 | 0.78 |
|  | LSAS | -0.06 | 0.049 | -.17 | .253 | -0.16 | 0.06 |
|  | AQ | -0.11 | 0.263 | -.13 | .687 | -0.67 | 0.23 |
|  | TAS-20 | 0.29 | 0.13 | .37 | .040* | 0.02 | 0.60 |
|  | AP | 0.49 | 8.874 | .02 | .961 | -15.06 | 13.64 |
|  | NAP | -5.50 | 12.96 | -.20 | .597 | -30.98 | 14.75 |
|  | AP x AQ | 0.40 | 0.368 | .62 | .251 | -0.31 | 1.43 |
|  | NAP x AQ | 0.58 | 0.416 | .74 | .152 | -0.37 | 1.53 |
|  | AP x TAS-20 | -0.28 | 0.225 | -.72 | .225 | -0.69 | 0.07 |
|  | NAP x TAS-20 | -0.14 | 0.324 | -.31 | .632 | -0.75 | 0.54 |

AP: Autistic participants; NAP: Non-autistic participants with difficulties in social interaction.

Neurotypical participants were used as reference group.

*Indicates *p* < .05. **Indicates *p* < .01. ***Indicates *p* < .001.

**Table S5a**

*Linear models of predictors of avoidant PD dimension in males. 95 % bias corrected and accelerated confidence intervals. Confidence intervals and standard errors based on 1,000 bootstrap samples.*

| Models | Predictors | *b* | *SE B* | *β* | *p* | BCa 95%  Confidence interval | |
| --- | --- | --- | --- | --- | --- | --- | --- |
|  |  |  |  |  |  | Lower | Upper |
| 1 | (Constant) | 4.99 | 2.96 |  | .085 | -0.46 | 10.53 |
|  | Age | 0.02 | 0.06 | .02 | .750 | -0.11 | 0.12 |
|  | BDI-II | 0.27 | 0.06 | .29 | <.001*** | 0.15 | 0.39 |
|  | LSAS | 0.15 | 0.03 | .46 | <.001*** | 0.09 | 0.21 |
|  | AQ | 0.09 | 0.08 | .10 | .256 | -0.06 | 0.26 |
|  | TAS-20 | 0.13 | 0.07 | .15 | .069 | -0.01 | 0.26 |
| 2 | (Constant) | -0.50 | 8.07 |  | .951 | -14.34 | 19.63 |
|  | Age | 0.04 | 0.06 | .04 | .549 | -0.08 | 0.15 |
|  | BDI-II | 0.24 | 0.06 | .27 | <.001*** | 0.11 | 0.38 |
|  | LSAS | 0.15 | 0.03 | .47 | <.001*** | 0.10 | 0.21 |
|  | AQ | 0.40 | 0.26 | .42 | .110 | -0.17 | 0.81 |
|  | TAS-20 | 0.11 | 0.16 | .13 | .468 | -0.21 | 0.37 |
|  | AP | 10.92 | 9.58 | .53 | .267 | -7.54 | 25.62 |
|  | NAP | 2.04 | 10.50 | .08 | .838 | -18.28 | 16.65 |
|  | AP x AQ | -0.53 | 0.29 | -.96 | .072 | -1.07 | 0.15 |
|  | NAP x AQ | -0.21 | 0.29 | -.28 | .486 | -0.78 | 0.50 |
|  | AP x TAS-20 | 0.04 | 0.18 | .12 | .827 | -0.32 | 0.47 |
|  | NAP x TAS-20 | 0.03 | 0.19 | .07 | .858 | -0.36 | 0.45 |

AP: Autistic participants; NAP: Non-autistic participants with difficulties in social interaction.

Neurotypical participants were used as reference group.

*Indicates *p* < .05. **Indicates *p* < .01. ***Indicates *p* < .001.

**Table S5b**

*Linear models of predictors of avoidant PD traits in females. 95 % bias corrected and accelerated confidence intervals. Confidence intervals and standard errors based on 1,000 bootstrap samples.*

| Models | Predictors | *b* | *SE B* | *β* | *p* | BCa 95%  Confidence interval | |
| --- | --- | --- | --- | --- | --- | --- | --- |
|  |  |  |  |  |  | Lower | Upper |
| 1 | (Konstante) | 4.57 | 3.07 |  | .131 | -0.80 | 9.98 |
|  | Age | -0.02 | 0.07 | -.01 | .791 | -0.15 | 0.12 |
|  | BDI-II | 0.10 | 0.10 | .10 | .385 | -0.09 | 0.28 |
|  | LSAS | 0.16 | 0.04 | .48 | <.001*** | 0.09 | 0.24 |
|  | AQ | 0.21 | 0.09 | .25 | .017* | 0.03 | 0.37 |
|  | TAS-20 | 0.09 | 0.08 | .12 | .247 | -0.07 | 0.26 |
| 2 | (Konstante) | 2.76 | 5.30 |  | .609 | -7.10 | 10.76 |
|  | Age | 0.01 | 0.07 | .00 | .951 | -0.18 | 0.21 |
|  | BDI-II | 0.09 | 0.10 | .09 | .410 | -0.10 | 0.31 |
|  | LSAS | 0.14 | 0.04 | .40 | .002** | 0.06 | 0.22 |
|  | AQ | 0.15 | 0.17 | .17 | .355 | -0.23 | 0.42 |
|  | TAS-20 | 0.16 | 0.11 | .20 | .183 | -0.07 | 0.40 |
|  | AP | 11.16 | 11.42 | .44 | .312 | -11.65 | 38.62 |
|  | NAP | 4.89 | 9.85 | .18 | .624 | -15.29 | 20.77 |
|  | AP x AQ | 0.41 | 0.44 | .64 | .273 | -0.79 | 1.22 |
|  | NAP x AQ | 0.01 | 0.23 | .01 | .977 | -0.44 | 0.58 |
|  | AP x TAS-20 | -0.41 | 0.23 | -1.03 | .080 | -0.81 | 0.01 |
|  | NAP x TAS-20 | -0.02 | 0.17 | -.05 | .910 | -0.31 | 0.26 |

AP: Autistic participants; NAP: Non-autistic participants with difficulties in social interaction.

Neurotypical participants were used as reference group.

*Indicates *p* < .05. **Indicates *p* < .01. ***Indicates *p* < .001.

**Table S6a**

*Linear models of predictors of OCPD traits in males. 95 % bias corrected and accelerated confidence intervals. Confidence intervals and standard errors based on 1,000 bootstrap samples.*

| Models | Predictors | *b* | *SE B* | *β* | *p* | BCa 95%  Confidence interval | |
| --- | --- | --- | --- | --- | --- | --- | --- |
|  |  |  |  |  |  | Lower | Upper |
| 1 | (Constante) | 14.63 | 3.72 |  | .003** | 7.06 | 22.02 |
|  | Age | -0.01 | 0.07 | -.01 | .883 | -0.14 | 0.13 |
|  | BDI-II | 0.07 | 0.07 | .08 | .316 | -0.06 | 0.20 |
|  | LSAS | 0.08 | 0.03 | .28 | .008** | 0.02 | 0.14 |
|  | AQ | 0.34 | 0.08 | .39 | <.001*** | 0.19 | 0.48 |
|  | TAS-20 | 0.00 | 0.08 | .00 | .981 | -0.16 | 0.16 |
| 2 | (Constant) | 15.44 | 11.66 |  | .189 | -3.25 | 43.06 |
|  | Age | 0.00 | 0.07 | .00 | .965 | -0.13 | 0.13 |
|  | BDI-II | 0.08 | 0.08 | .10 | .306 | -0.07 | 0.21 |
|  | LSAS | 0.09 | 0.03 | .32 | .003** | 0.03 | 0.16 |
|  | AQ | 0.55 | 0.35 | .63 | .113 | -0.25 | 1.06 |
|  | TAS-20 | -0.08 | 0.20 | -.11 | .660 | -0.41 | 0.19 |
|  | AP | -8.32 | 12.83 | -.44 | .515 | -34.57 | 11.07 |
|  | NAP | -3.41 | 14.48 | -.15 | .805 | -30.97 | 16.95 |
|  | AP x AQ | -0.16 | 0.38 | -.31 | .677 | -0.80 | 0.75 |
|  | NAP x AQ | -0.14 | 0.42 | -.21 | .732 | -0.89 | 0.78 |
|  | AP x TAS-20 | 0.14 | 0.21 | .47 | .498 | -0.28 | 0.71 |
|  | NAP x TAS-20 | 0.07 | 0.25 | .20 | .746 | -0.48 | 0.81 |

AP: Autistic participants; NAP: Non-autistic participants with difficulties in social interaction.

Neurotypical participants were used as reference group.

*Indicates *p* < .05. **Indicates *p* < .01. ***Indicates *p* < .001.

**Table S6b**

*Linear models of predictors of OCPD traits in females. 95 % bias corrected and accelerated confidence intervals. Confidence intervals and standard errors based on 1,000 bootstrap samples.*

| Models | Predictors | *b* | *SE B* | *β* | *p* | BCa 95%  Confidence interval | |
| --- | --- | --- | --- | --- | --- | --- | --- |
|  |  |  |  |  |  | Lower | Upper |
| 1 | (Constant) | 14.03 | 3.46 |  | <.001*** | 7.81 | 21.68 |
|  | Age | 0.04 | 0.07 | .04 | .621 | -0.11 | 0.16 |
|  | BDI-II | 0.24 | 0.09 | .29 | .010* | 0.06 | 0.40 |
|  | LSAS | 0.06 | 0.04 | .21 | .089 | -0.01 | 0.14 |
|  | AQ | 0.20 | 0.09 | .26 | .026* | 0.02 | 0.38 |
|  | TAS-20 | 0.03 | 0.08 | .05 | .656 | -0.13 | 0.21 |
| 2 | (Constant) | 11.46 | 6.40 |  | .083 | 0.49 | 23.84 |
|  | Age | 0.06 | 0.08 | .06 | .468 | -0.10 | 0.19 |
|  | BDI-II | 0.20 | 0.10 | .24 | .060 | 0.02 | 0.37 |
|  | LSAS | 0.08 | 0.04 | .27 | .056 | 0.00 | 0.19 |
|  | AQ | 0.47 | 0.20 | .63 | .027* | 0.11 | 1.02 |
|  | TAS-20 | -0.01 | 0.12 | -.01 | .964 | -0.23 | 0.23 |
|  | AP | -3.58 | 11.73 | -.16 | .744 | -27.02 | 16.32 |
|  | NAP | -8.35 | 10.07 | -.34 | .348 | -33.09 | 21.98 |
|  | AP x AQ | -0.20 | 0.39 | -.35 | .574 | -0.91 | 0.50 |
|  | NAP x AQ | -0.39 | 0.29 | -.55 | .162 | -0.93 | -0.04 |
|  | AP x TAS-20 | 0.06 | 0.25 | .17 | .806 | -0.47 | 0.62 |
|  | NAP x TAS-20 | 0.27 | 0.18 | .66 | .119 | -0.06 | 0.58 |

AP: Autistic participants; NAP: Non-autistic participants with difficulties in social interaction.

Neurotypical participants were used as reference group.

*Indicates *p* < .05. **Indicates *p* < .01. ***Indicates *p* < .001.
